# *In vitro* ability of beer fermentation residue and yeast-based products to bind aflatoxin B_1_

**DOI:** 10.1590/S1517-838246220130400

**Published:** 2015-06-01

**Authors:** Fernanda Bovo, Larissa Tuanny Franco, Roice Eliana Rosim, Ricardo Barbalho, Carlos Augusto Fernandes de Oliveira

**Affiliations:** 1 Universidade de São Paulo, Departamento de Engenharia de Alimentos, Faculdade de Zootecnia e Engenharia de Alimentos, Universidade de São Paulo, Pirassununga, SP, Brasil, Departamento de Engenharia de Alimentos, Faculdade de Zootecnia e Engenharia de Alimentos, Universidade de São Paulo, Pirassununga, SP, Brazil.; 2 ICC Brazil, São Paulo, SP, Brasil, ICC Brazil, São Paulo, SP, Brazil.

**Keywords:** AFB_1_, binding capacity, decontamination, *S. cerevisiae* cells

## Abstract

This study aimed to verify the *in vitro* ability of beer fermentation residue (BFR) containing *Saccharomyces cerevisiae* cells and five commercial products that differed in the viability and integrity of *S. cerevisiae* cells to remove aflatoxin B_1_ (AFB_1_) from a citrate-phosphate buffer solution (CPBS). BFR was collected at a microbrewery and prepared by drying and milling. The commercial yeast-based products were as follows: inactive intact yeast cells from beer alcoholic fermentation, inactive intact yeast cells from sugarcane alcoholic fermentation, hydrolyzed yeast cells, yeast cell walls and active yeast cells. Adsorption assays were performed in CPBS spiked with 1.0 μg AFB_1_/mL at pH 3.0 and 6.0 for a contact time of 60 min at room temperature. Analysis of AFB_1_ in the samples was performed by high performance liquid chromatography. AFB_1_ adsorption by the products ranged from 45.5% to 69.4% at pH 3.0 and from 24.0% to 63.8% at pH 6.0. The higher percentages (p < 0.05) of AFB_1_ binding at both pH values were achieved with products containing hydrolyzed yeast cells or yeast cell walls rather than intact cells. The AFB_1_ binding percentages of BFR were 55.0 ± 5.0% at pH 3.0 and 49.2 ± 4.5% at pH 6.0, which was not significantly different (p > 0.05) from commercial products containing inactive intact yeast cells. The results of this trial indicate that the yeast-based products tested, especially the BFR, have potential applications in animal feeds as a suitable biological method for reducing the adverse effects of aflatoxins.

## Introduction

Aflatoxin B_1_ (AFB_1_) is the main metabolite produced by fungi of the genus *Aspergillus*, primarily *A. flavus* and *A. parasiticus*, which can contaminate corn, sorghum, oilseeds, and other food materials (An, 2005). Various domestic and experimental species are sensitive to the carcinogenic, mutagenic, hepatotoxic and immunosuppressive effects of AFB_1_, in which the liver is the main affected organ (Hussein and Brasel, 2001).

Concern about the negative impacts of AFB_1_ on animal health and the agricultural economy led to an investigation of strategies to prevent their formation, as well as to eliminate, inactivate or reduce their bioavailability in contaminated products (Hernandez-Mendoza *et al.*, 2009). Enteroadsorption methods use nutritionally inert dietary compounds that prevent toxin absorption by the animal gastrointestinal tract (Gratz *et al.*, 2005). Although several mineral adsorbents are available, their application is limited due to vitamin and mineral adsorption (Hussein and Brasel, 2001). An attractive alternative is the use of microorganisms to control or eliminate aflatoxins (AF) in food and feed, thus preserving their quality and safety (Alberts *et al.*, 2009).

The adsorptive capacity of yeast cells has been widely studied, and yeast is a promising candidate for AF decontamination (Aravind *et al.*, 2003; Fruhauf *et al.*, 2012; Stanley *et al.*, 2004). Commercial products made from either whole yeast cells or partial yeast cells, such as cell wall glucans and mannans, are used in animal rations not only to facilitate toxin adsorption but also to improve productivity parameters. Unlike the yeast cells used in ethanol fuel production from sugar cane, which are often reused throughout the season, the yeast cells used in beer production are discharged at the end of each process. In addition to its excellent nutritional value, yeast biomass from beer fermentation has been hypothesized to be a viable alternative for removing AF, especially considering its wide availability both worldwide and in Brazil. Therefore, the objective of this study was to perform *in vitro* tests to verify the ability of beer fermentation residue to remove AFB_1_ from a citrate-phosphate buffer solution and to then compare the results with those found for five commercial products made from yeast cells.

## Materials and Methods

### Yeast cell based products

Beer fermentation residue (BFR) containing cells of *Saccharomyces cerevisiae* was obtained from a microbrewery located in the State of São Paulo, Brazil. BFR was collected and transported to the Laboratory of Food Microbiology and Mycotoxicology (College of Animal Science and Food Engineering, University of São Paulo), and the number of yeast cells was counted using a Neubauer chamber (BOE13 - Boeco, Hamburg, Germany). Convenient volumes of BFR (4.0 L) containing 10^10^ cells/mL were transferred to aluminum pans for drying in an oven with forced air circulation (320-SE - Fanem, São Paulo, SP, Brazil) at 100 °C to constant weight to obtain a dry mass of BFR containing *S. cerevisiae* cells. The dried residue was ground in a mill (TE-631/2 - Tecnal, Piracicaba, SP, Brazil) and stored at room temperature for further use in the *in vitro* AFB_1_ adsorption assays.

Adsorption tests of AFB_1_ were also performed using five commercial yeast-based products containing *S. cerevisiae* cells or part of these cells, as described in Table 1. Products numbered from 2 to 5 were produced and kindly donated by ICC Brazil (São Paulo, SP, Brazil). Product number 6 (SAFLAGER W37/70) was acquired from Fermentis Ltd. (Marcq en Baroeul, France).

**Table 1:** Yeast-based products containing cells of *S. cerevisiae* used in the AFB_1_ adsorption assays.

Product	Identification	Description
1	BFR^1^	Residue from beer alcoholic fermentation
2	BFIY^2^	Inactive yeast cells from beer alcoholic fermentation
3	SFIY^2^	Inactive yeast cells from sugarcane alcoholic fermentation
4	Hilyses^2^	Hydrolyzed yeast cells
5	ImmunoWall^2^	Yeast cell walls
6	Saflager^3^	Active yeast cells from beer alcoholic fermentation

1 Beer fermentation residue collected at a microbrewery in São Paulo, Brazil, and prepared in the laboratory by drying and milling.

2 Yeast-based products produced by ICC Brazil (São Paulo, SP, Brazil).

3 Yeast *S. cerevisiae* SAFLAGER W37/70 produced by Fermentis Ltd. (Marcq en Baroeul, France).

### Adsorption Assays of AFB_1_

A standard of AFB_1_ (Supelco, Bellefonte, PA, USA) was dissolved in toluene and acetonitrile (9:1), calibrated in a spectrophotometer (Spectrumlab 22PC - Shanghai Lengguang Technology Co. Ltd, Shanghai, China) according to Scott (1990), and diluted to obtain a stock solution containing approximately 10.0 μg AFB_1_/mL. The previous solution was used in the preparation of other working solutions containing approximately 1.0 μg AFB_1_/mL in a citrate-phosphate buffer solution (pH 3.0 and pH 6.0) prepared using a combination of solutions of 0.1 M citric acid (Synth, Diadema, SP, Brazil) and 0.2 M bibasic sodium phosphate (Süd Chemie, Jacareí, SP, Brazil). The solvent was completely evaporated by direct injection of air over a heating bath at 40 °C (TE-019 -Tecnal, Piracicaba, SP, Brazil).

The assays evaluating the efficiency of adsorbents to remove AFB_1_ from a contaminated medium were conducted at pH of 3.0 and 6.0 according to Ledoux and Rottinghaus (1999). For each pH, 0.05 g of each sample was weighed and suspended in 5 mL of buffer solution (pH 3.0 or pH 6.0) spiked with AFB_1_ and incubated in an orbital shaker (TE-140 - Tecnal, Piracicaba, SP, Brazil) at 180 rpm for 60 min at room temperature. Following this step, centrifugation was performed at 1,800 × g for 10 min (CT-14000 - Cientec, Piracicaba, SP, Brasil), and then 2 mL of the supernatant was collected and stored at −20 °C for a subsequent injection into the High Performance Liquid Chromatography (HPLC) system. The assays were performed in triplicate, and we also incubated and analyzed a positive (AFB_1_ in buffer solution) and negative (0.05 g of sample in buffer solution) control.

### Quantification of AFB_1_ by HPLC

AFB_1_ quantification in the buffer solutions was achieved by direct injection into a Shimadzu HPLC (Tokyo, Japan) system consisting of a fluorescence detector (RF-10A XL) and an autosampler (SIL-10AF). An ODS column 5 μm 4.6 × 150 mm (Phenomenex, Torrance, CA, USA) was used. The system was stabilized for one hour at a flow rate of 1 mL/min at room temperature. The mobile phase was a solution of water, acetonitrile and methanol (60:20:20) at a flow rate of 1 mL/min. The excitation detection was performed at a wavelength of 360 nm, and emission was monitored at 440 nm. Under the above conditions, the detection limit for AFB_1_ was 0.01 ng/mL, and the retention time was approximately 10.5 min with a retention window of ± 10%.

The quantification of the percentage of AFB_1_ adsorbed was performed using Eq. 1, where A represents the percentage of AFB_1_ adsorbed by the sample, B the area of positive control chromatographic peak (AFB_1_ in buffer solution), C the area of sample chromatographic peak (AFB_1_ in buffer solution + sample) and D the area of negative control chromatographic peak (buffer solution + sample).

(1) $$A=\frac{B-C-D}{B}*100$$

### Statistical analysis

The results were subjected to ANOVA in accordance with the procedures established in the General Linear Model of SAS (1992) to assess significant differences between the means of variables in the different treatments. For comparison between means, we used the Fisher LSD test and adopted a rejection level of α = 0.05.

## Results and Discussion

The results obtained for AFB_1_ adsorption efficiency by BFR and commercial yeast-based products in a contaminated medium are presented in Table 2. The percentages of toxin adsorption by all products ranged from 45.5% to 69.4% at pH 3.0 (p < .0001) and from 24.0% to 63.8% at pH 6.0 (*P* < .0001). The Hilyses and ImmunoWall products had the best capacity to adsorb AFB_1_ at both pH values and were quite similar (p > 0.05). BFR bound the toxin at 55.0% at pH 3.0 and 49.2% at pH 6.0, which did not differ significantly from products BFIY and SFIY but did have greater values than the Saflager product at pH 3.0 or 6.0. Only products BFIY (p = 0.0212), Hilyses (p = 0.0256) and Saflager (p = 0.0003) differed significantly when compared at different pH values.

**Table 2:** AFB_1_ adsorption results of *S. cerevisiae* cell based products in citrate-phosphate buffer solution.

Products^1^	Aflatoxin B_1_ Adsorbed^2^ (%)		p value
		
	pH 3.0	pH 6.0	
BFR	55.0 ± 5.0 b	49.2 ± 4.5 b	0.2096
BFIY	56.3 ± 4.2 b	45.9 ± 2.5 b	0.0212
SFIY	53.2 ± 2.0 b	49.7 ± 2.6 b	0.1351
Hilyses	69.4 ± 0.9 a	60.0 ± 4.6 a	0.0256
ImmunoWall	66.7 ± 2.6 a	63.8 ± 1.2 a	0.1578
Saflager	45.5 ± 2.8 c	24.0 + 1.3 c	0.0003

1 BFR: Inactive yeast cells from beer alcoholic fermentation dried and milled; BFIY: Inactive yeast cells from beer alcoholic fermentation; SFIY: Inactive yeast cells from sugarcane alcoholic fermentation; Hilyses: Hydrolyzed yeast cells; ImmunoWall: Yeast cell walls; Saflager: Active yeast cells from beer alcoholic fermentation.

2 Values expressed as mean ± standard deviation of samples analyzed in triplicate.

a–c Within a column, means without a common superscript differ significantly (p < 0.05).

Jouany *et al.* (2005) explained that the *S. cerevisiae* cell wall is composed mainly of polysaccharides (80–90%) and that their mechanical strength is due to an inner layer formed by chains of β-D-glucans. These β-D-glucans are composed of a complex network of β-(1,3)-D-glucans with a high degree of polymerization branched with β-(1,6)-D-glucans with a low degree of polymerization. This inner layer is firmly bound to the plasma membrane by linear chains of chitin, which has a significant role in the insolubility of the overall structure of the cell and in the packaging of β-D-glucans, both of which influence the plasticity of the cell wall. The outer layer of the yeast cell wall is composed of mannoprotein, which plays an important role in gas and nutrient exchange with the outside environment. The entire structure is highly dynamic and can vary with the yeast strain, phase of cell cycle, and growth conditions such as pH, temperature, oxygenation rate, medium nature and carbon source. Thus, such differences in cell wall composition among yeast strains may have influenced the ability of the tested products to bind AFB_1_ in the present study because all products included *S. cerevisiae* cells in their composition but were composed of different strains of the same yeast species.

β-D-glucans are the cell wall components responsible for complexation with the toxin, and the reticular organization of β-D-glucans and their distribution among β-(1,3) and β-(1 6)-D-glucans plays an important role in this efficacy. In addition, weak hydrogen bonds and van der Waals bonds are involved in the complex chemical formation between mycotoxins and β-D-glucans, leading to a chemical interaction of "adsorption" rather than "contact". Regarding AFB_1_, the toxin is bound to the glucans due to the interaction between the aromatic ring and the lactone and ketone groups of the polar form of AFB_1,_ as well as by chemical bonds with glucose units of the single helix β-D-glucans. Thus, the separation of the yeast cell wall from other cellular components, such as the cytoplasm and organelles, or the hydrolysis of the cell may expose a greater number of β-D-glucan units that were not previously available when the yeast cell was intact (Jouany *et al.*, 2005). These structural changes cause an increase in mycotoxin removal from the medium, which could possibly explain why the products Hilyses and ImmunoWall had a greater degree of AFB_1_ removal at pH 3.0 and 6.0. However, Hernandez-Mendoza *et al.* (2009) found that the integrity of the bacterial cell wall plays an important role in the process of AF removal by either viable or non-viable cells. In their study it was shown that both the bacterial cell wall and its purified fragments were able to remove the AF from the medium; however, when the cell wall was lost or destroyed due to enzymatic treatments, a significant reduction in removal capacity was observed.

Fruhauf *et al.* (2012) analyzed 30 commercial products composed of different concentrations of yeast cell walls and inorganic compounds and found that a higher ash content corresponded to lower mannan-oligosaccharide and β-glucan content. The authors concluded that the effectiveness of AFB_1_ removal was related to the ash content because eight products with over 30% of ash had toxin adsorption values over 90%, while eight of ten products with less than 10% ash showed adsorption rates lower than 25% in all tested mediums (pH 3.0 and 6.0 and gastric juice).

As mentioned before, we observed significant differences between the values of AFB_1_ adsorption at pH 3.0 and 6.0 for the BFIY, Hilyses and Saflager products. Raju and Devegowda (2002) did not observe differences between pH values when using esterified glucomannan, a yeast cell wall derivative, at a rate of 0.1% for AFB_1_ removal (300 ppb) (80.7% and 82.5%, pH 3.0 and 6.0, respectively). Diaz *et al.* (2002) found no differences in the removal of AFB_1_ by esterified glucomannan (96.6%) between the pH values analyzed (pH 3, 7, and 10; pH not adjusted after the addition of 1% of the product).

Using intact and viable cells of *S. cerevisiae*, Armando *et al.* (2011) observed percentages of AFB_1_ (500 ng/mL) removal between 20.2 and 65.5%, depending on the yeast strain. Shetty *et al.* (2007) analyzed 18 strains of viable *S. cerevisiae* from fermented corn dough and sorghum beer production and found that seven strains removed 10–20% of AFB_1_, 8 strains removed 20–40% and three strains removed more than 40%, again emphasizing the importance of the strain. These results were similar to those found in our study for the intact and viable cells present in the product Saflager (45.5% and 24.0%, respectively for pH 3.0 and 6.0).

Products containing inactive and intact yeast cells (BFIY, SFIY and BFR) presented greater values for AFB_1_ adsorption than the product containing active and intact yeast cells (Saflager). Cellular non-viability obtained by heating may increase the permeability of the outer yeast cell wall due to both the suspension of mannans from the cell surface and many physical and chemical changes, leading to increased availability of previously hidden binding sites. The continuity of AF removal, even after application of heat treatments, confirms once more that yeast cell viability is not a significant factor in the removal of toxins from the medium (Rahaie *et al.*, 2010).

The results of this trial indicated that all the tested yeast-based products containing cells of *S. cerevisiae* have the ability to partially remove AFB_1_ *in vitro*. The binding process was dependent on the conditions in which yeast cells were produced, including the viability and integrity of these cells as well as the specificity of each strain. Thus, we conclude that the yeast-based products tested, especially the BFR, have a potential application in animal feeds as a suitable biological method for reducing the adverse effects of AF. However, additional *in vivo* experiments are needed to confirm the viability of using BFR and other yeast-based products as adsorbents in animal feeds.
